# Ureteroureterostomy With Near-Infrared Ray Catheter in a Kidney Transplant

**DOI:** 10.7759/cureus.57687

**Published:** 2024-04-05

**Authors:** Satoshi Takai, Hayato Nishida, Hiroki Fukuhara, Masayuki Kurokawa, Norihiko Tsuchiya

**Affiliations:** 1 Department of Urology, Yamagata University Faculty of Medicine, Yamagata, JPN; 2 Department of Urology, Jichi Medical University Faculty of Medicine, Shimotsuke, JPN

**Keywords:** surgical therapy, ureteroureterostomy, near-infrared ray catheter, kidney transplantation, ureteral stenosis

## Abstract

Transplant ureteral stenosis (US) is a complication of kidney transplantation (KT) that sometimes adversely affects kidney function. Endoscopic treatment may be selected as the initial treatment; however, the recurrence rate is high. Ureteral reconstruction is necessary as a secondary treatment, but it is often difficult to identify the transplanted ureter due to reoperation; therefore, transplanted ureter and renal arteriovenous injury are intraoperative complications that should be noted. The Near-Infrared Ray Catheter (NIRC™) fluorescent ureteral catheter (NIRFUC) fluoresces by illuminating near-infrared rays, facilitating the identification of intraoperative ureteral locations.

Herein, we report the case of a 34-year-old woman who developed US following KT. She underwent balloon dilation for transplant US, but the stenosis recurred; therefore, she underwent transplant ureteral auto-ureteral anastomosis. Although it was difficult to identify and detach the transplanted ureter owing to adhesions, the use of NIRFUC facilitated the identification of the ureter in the surgical field and enabled safe end-side anastomosis between the transplanted ureter and the autologous ureter.

In conclusion, although there is no consensus on the best method for complex transplantation-related US cases, NIRFUC may be used to safely identify and perform surgeries on the ureter.

## Introduction

Ureteral stenosis (US) is a complication of kidney transplantation (KT) that adversely affects postoperative renal function and occurs at a frequency of 3.1-7.6% [1-4]. Although endoscopic treatment is often attempted first, its efficacy is approximately 40-61% [5,6], and surgical therapy is used as a secondary approach. However, identification and dissection of the grafted ureter is difficult because of reoperation, and the most important complication of reconstructive surgery is damage to the grafted ureter and renal arteriovenous vessels.

The Near-Infrared Ray Catheter (NIRC™) fluorescent ureteral catheter (NIRFUC) contains a special fluorescent dye that fluoresces when used with a near-infrared camera. It has been used for intraoperative ureteral navigation mainly in gastrointestinal surgery and gynecology [7,8].

Herein, we report a case of a 34-year-old woman who was found to have US after KT. After endoscopic treatment failed to improve her US, we used NIRFUC intraoperatively to safely identify the transplanted ureter and perform the ureteroureterostomy.

## Case presentation

**Figure 1:**
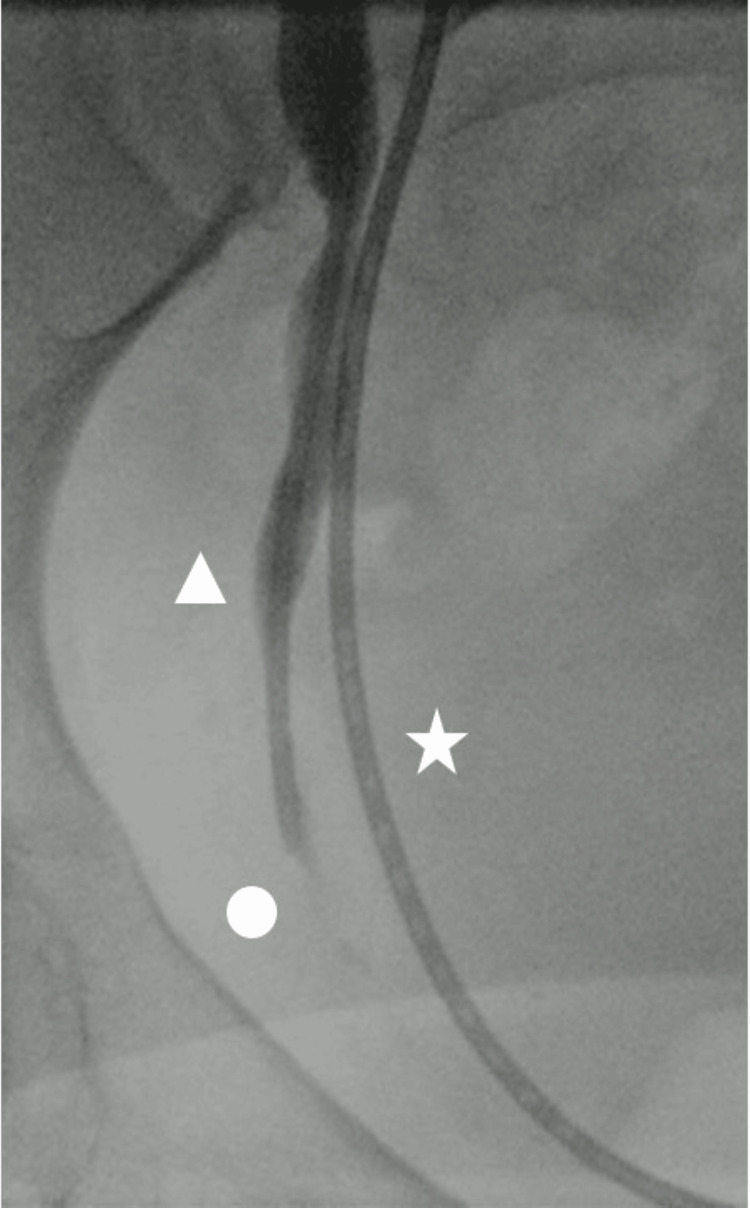
Fluoroscopic image of the right pelvic cavity is shown. Fluoroscopic image showing the area of the transplanted US (circle). NIRFUC is retrogradely implanted into the autologous ureter (star). Progressively, NIRFUC is placed just above the transplanted US (triangle). US: ureteral stenosis; NIRFUC: Near-Infrared Ray Catheter (NIRC™) fluorescent ureteral catheter

**Figure 2:**
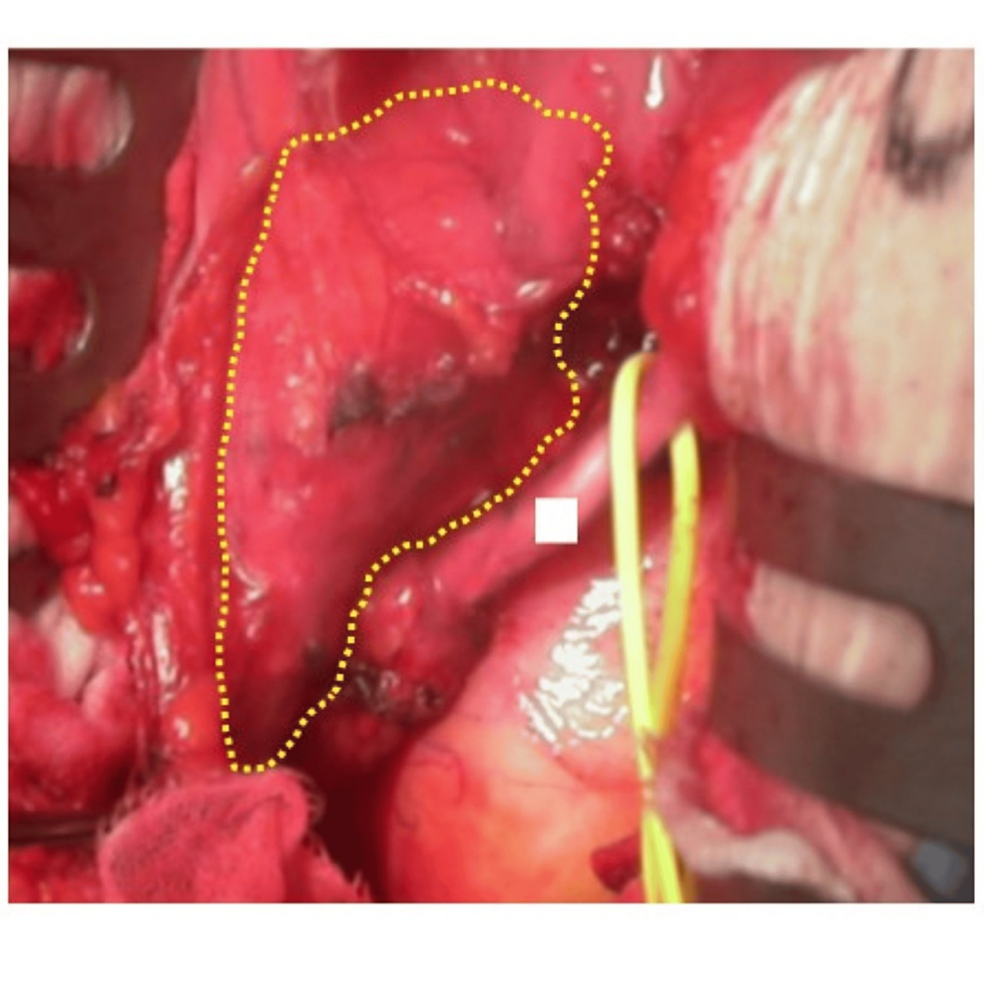
Intraoperative photographs before near-infrared irradiation are shown. The autologous ureter could be identified (square), but the transplanted ureter was difficult to identify and detach due to strong adhesion to the surrounding area (the area within the dotted lines).

**Figure 3:**
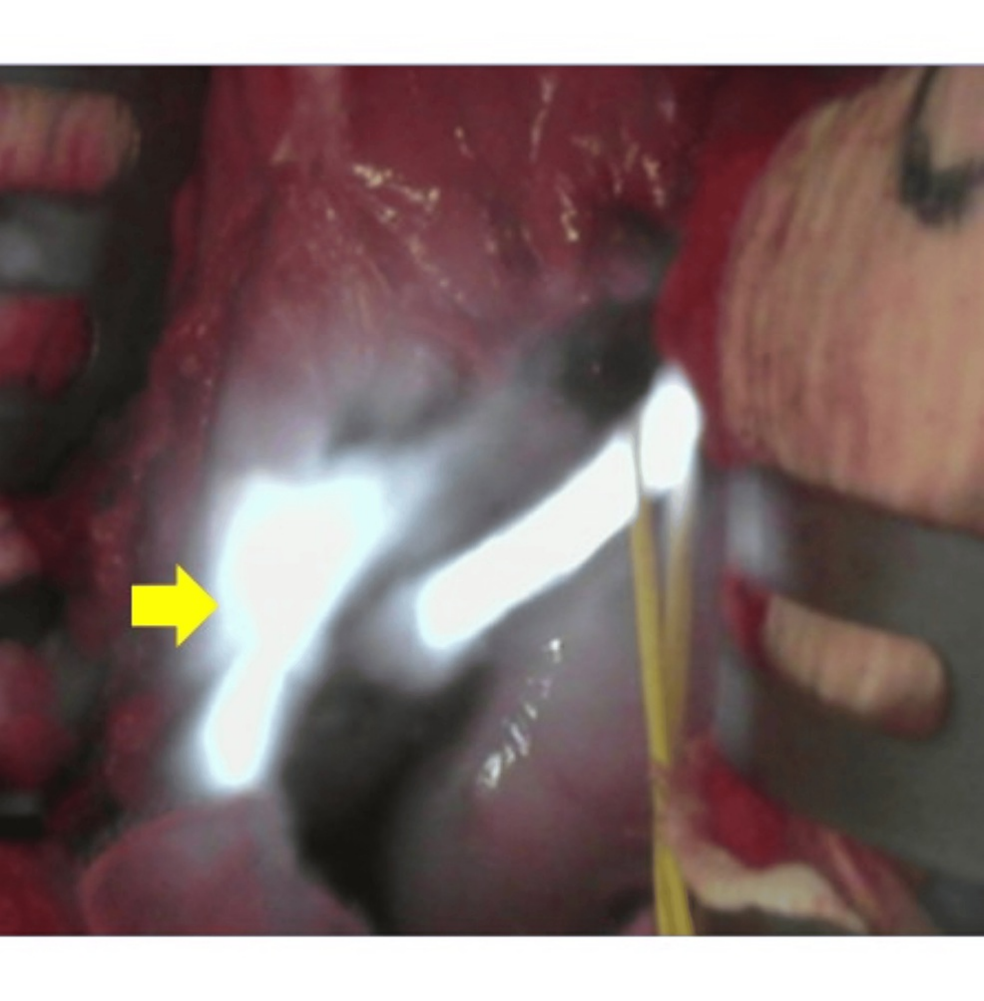
Intraoperative photographs after near-infrared irradiation are shown. NIRFUC fluorescence facilitated the identification of the transplanted ureter (arrow). NIRFUC: Near-Infrared Ray Catheter (NIRC™) fluorescent ureteral catheter

**Figure 4:**
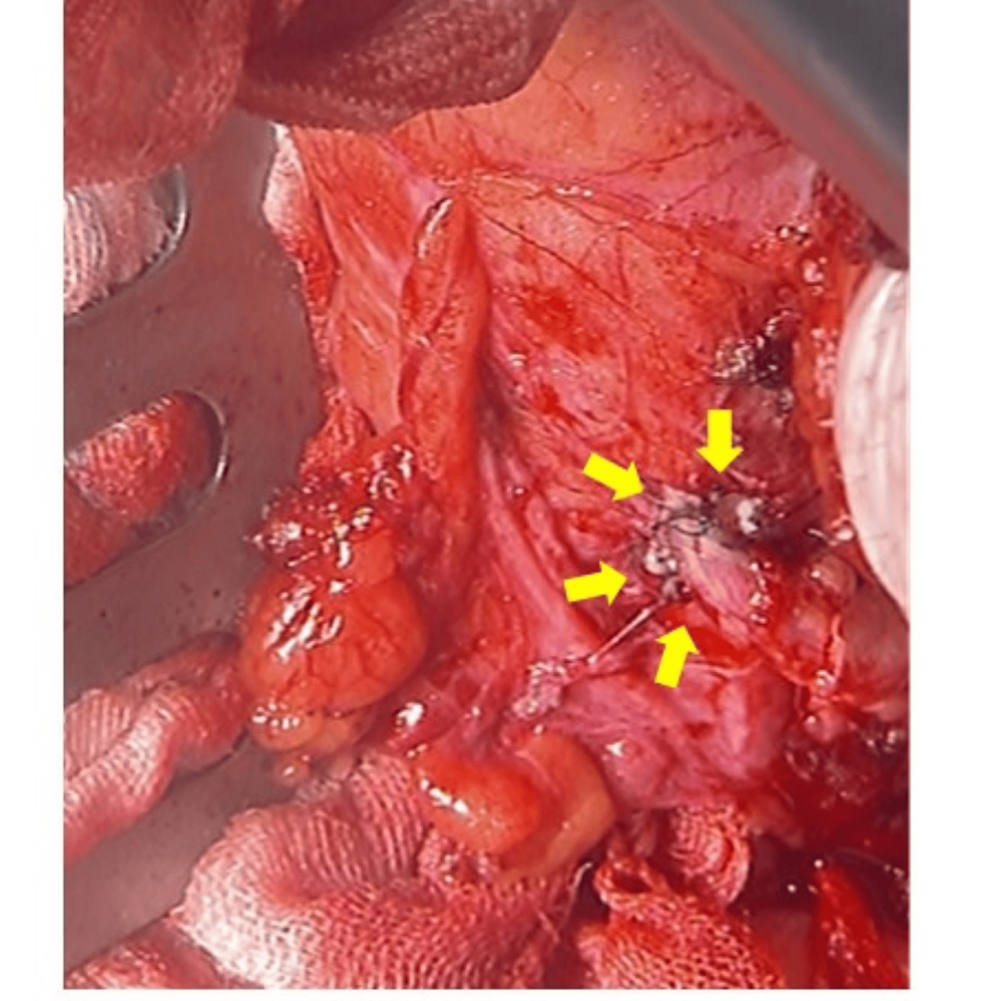
Intraoperative photographs are shown after the transplant ureteral auto-ureteral anastomosis was performed. Safe end-to-side anastomosis was achieved (arrows).

A 34-year-old woman who had been on dialysis for eight years and three months for chronic renal failure due to diabetic nephropathy underwent an ABO-incompatible living donor KT, with her father as the donor. At the time of surgery, stones in the donor kidney, which were detected using computed tomography (CT) before surgery, were extracted on the back table. Two months after KT, ultrasound examination and CT showed hydronephrosis of the transplanted kidney, stenosis of the transplanted renal ureter, and ureteral calculus at the site of stenosis. Six months after KT, transurethral lithotripsy was attempted but was abandoned because the guidewire could not pass through the stenosis in a retrograde manner. We then performed a percutaneous nephrostomy. Seven months after KT, we performed balloon dilatation for US and lithotripsy for ureteral stones using the antegrade approach from the nephrostomy. However, pyelonephrography performed one week after surgery revealed US recurrence. Eight months after KT, the patient underwent autologous ureteral anastomosis of the transplanted ureter. In this surgery, a NIRFUC was used to identify both implanted and autologous ureters. After anesthesia induction, a NIRFUC was placed retrogradely into the autologous ureter. Subsequently, another NIRFUC was placed in the transplanted ureter through the nephrostomy, in front of the US (Figure 1). A skin incision was made along the wound during KT surgery, and the abdomen was opened. Identification of the autologous ureter was easy; however, the transplanted ureter was difficult to identify and detach due to severe adhesions that developed following KT surgery. The surgical field was then illuminated using near-infrared light; the NIRFUCs emitted light and the implanted ureter could be easily identified (Figure 2 and Figure 3). This allowed us to safely perform transplant ureteral auto-ureteral anastomosis without damaging the renal arteriovenous vessels and ureters (Figure 4). The postoperative course was uneventful, and no signs of worsening renal function or hydronephrosis were observed at the final follow-up one year postoperatively.

## Discussion

Although KT outcomes have improved in recent years, the incidence of urological complications remains high. Transplant US, which frequently deteriorates grafted kidney function, is one of the most common complications and occurs in approximately 3.1-7.6% of KT cases [1-4]. Non-intensive procedures such as balloon dilation of the stenosis or laser incision are recommended. Such endoscopic procedures are only 40-61% effective and may require multiple treatments [5,6]. Therefore, vesicoureteral reanastomosis or autologous ureteral anastomosis should be considered as a secondary approach, but the most important complication to be considered is damage to the transplanted ureter and surrounding vessels, including the grafted kidney and iliac vessels, due to difficulty in distinguishing the transplanted ureter from other luminal tissues owing to severe adhesions. In our case, balloon dilation was performed initially as the primary treatment, but restenosis soon occurred. Hence, surgical treatment was performed as a second-line treatment; however, as shown in Figure 2, it was very difficult to identify the transplanted ureter during laparotomy.

Liu et al. reported that open ureteral reconstruction was performed after an accurate diagnosis of stenosis using magnetic resonance urography (MRU) [1]. Kroczak et al. reported that the Boari flap procedure is safe in patients who experience recurrence after ureteral dilation or conventional open surgery [9,10]. Although surgical techniques have been developed, there is currently no consensus on the optimal method for complex transplant US cases.

Identifying the transplanted ureter safely and reliably is crucial to avoid surgical complications in this procedure. We developed a safe means of identifying the transplanted ureter using NIRFUC.

NIRFUC (Cardinal Health, Tokyo, Japan) is a catheter material composed of a dye with fluorescence properties similar to those of indocyanine green. The use of a near-infrared camera suitable for this purpose helps locate the ureter and reduces the risk of ureteral injury. There have been several reports suggesting the usefulness of fluorescent ureteral catheters for intraoperative navigation in cases with a high risk of adhesions and inflammation and in cases of reoperation due to postoperative ureteral obstruction, mainly in the fields of gastroenterology and gynecology, as well as for urethral identification in laparoscopic, robot-assisted, and open surgery [7,8,11-13]. The use of NIRFUC in open ureteral reconstruction after KT has not been previously reported in the literature. During the surgical procedure, it was difficult to identify the transplanted ureter due to severe adhesions; therefore, NIRFUC was used to enable visualization, and the procedure was performed successfully without any damage to the surrounding tissues. This underscores the usefulness of NIRFUC in open ureteral reconstruction.

Ultrasonography can also aid in ureteral identification when a hydroureter is present; however, it may not always be effective, depending on the position and angle of the probe and the skill of the surgeon. NIRFUC can be used in combination with ultrasonography to ensure visualization and identification of the ureter.

Although there is no consensus on the usefulness of preoperative ureteral catheterization, as in this case, even in open surgery, it may be difficult to palpate the catheter if the adhesions are severe. NIRFUC is particularly useful when there are concerns regarding severe adhesions around the transplanted ureter.

## Conclusions

Severe adhesion due to reoperation makes it difficult to identify the transplanted ureter during ureteral reconstruction for US following KT. We reported a case in which transplant ureteral auto-ureteral anastomosis was safely performed with NIRFUC for US after KT. Facilitating the identification of ureters using NIRFUC is an effective method for safely completing ureteroureterostomy and avoiding complications such as grafted kidney, iliac vessel, and transplant ureter injury.
